# What Exactly is Meant by “Loss of Domain” for Ventral Hernia? Systematic Review of Definitions

**DOI:** 10.1007/s00268-018-4783-7

**Published:** 2018-09-05

**Authors:** S. G. Parker, S. Halligan, S. Blackburn, A. A. O. Plumb, L. Archer, S. Mallett, A. C. J. Windsor

**Affiliations:** 1 The Abdominal Wall Unit, University College London Hospital, University College London Hospitals NHS Foundation Trust, 235 Euston Road, London, NW1 2BU UK; 2 UCL Centre for Medical Imaging, Charles Bell House, 43-45 Foley Street, Fitzrovia, London, W1W 7TS UK; 3 Institute of Applied Health Sciences, College of Medical and Dental Sciences, University of Birmingham, Edgbaston, Birmingham, B15 2TT UK

## Abstract

Large ventral hernias are a significant surgical challenge. “Loss of domain” (LOD) expresses the relationship between hernia and abdominal volume, and is used to predict operative difficulty and success. This systematic review assessed whether different definitions of LOD are used in the literature. The PubMed database was searched for articles reporting large hernia repairs that explicitly described LOD. Two reviewers screened citations and extracted data from selected articles, focusing on the definitions used for LOD, study demographics, study design, and reporting surgical specialty. One hundred and seven articles were identified, 93 full-texts examined, and 77 were included in the systematic review. Sixty-seven articles were from the primary literature, and 10 articles were from the secondary literature. Twenty-eight articles (36%) gave a written definition for loss of domain. These varied and divided into six broad groupings; four described the loss of the right of domain, six described abdominal strap muscle contraction, five described the “second abdomen”, five describing large irreducible hernias. Six gave miscellaneous definitions. Two articles gave multiple definitions. Twenty articles (26%) gave volumetric definitions; eight used the Tanaka method [hernia sac volume (HSV)/abdominal cavity volume] and five used the Sabbagh method [(HSV)/total peritoneal volume]. The definitions used for loss of domain were not dependent on the reporting specialty. Our systematic review revealed that multiple definitions of loss of domain are being used. These vary and are not interchangeable. Expert consensus on this matter is necessary to standardise this important concept for hernia surgeons.

**Electronic supplementary material:**

The online version of this article (10.1007/s00268-018-4783-7) contains supplementary material, which is available to authorized users.

## Introduction

The incidence of ventral hernia disease is increasing [1, 2]. This is due to an ageing population [3], the obesity epidemic [4], and an increasing number of abdominal operations being performed [5]. The proportion of complex ventral hernia (CVH) has also increased, partly due to the reasons already mentioned but also because of improvements in intensive care medicine [6]. In many patients’ following intra-abdominal sepsis and laparostomy, the ventral defect is left open and covered only via skin grafting, culminating in large ventral hernia. These CVH contain a significant proportion of the abdominal viscera outside the abdominopelvic compartment and their repair presents the sternest surgical challenge. “Loss of domain” (LOD) is a term used commonly in the hernia literature to describe the distribution of abdominal content between the hernia and residual abdominopelvic cavity. After repairing hernias with significant LOD (i.e. large hernias with much of the abdominal viscera outside the abdominal compartment), serious physiological complications can arise. The increase in intra-abdominal pressure pushes up on the diaphragm and can cause respiratory failure and pneumonia. The rise in abdominal pressure increases the tension along the laparotomy incision, which can be pulled apart resulting in wound complications [7] and hernia recurrence [7]. Post-operative recurrence is a significant problem and has precipitated interest in discerning pre-operative factors that help to predict success or failure [8, 9]. LOD may have prognostic value, and accordingly a standardised definition is warranted. A standardised definition will allow for comparable pre-operative assessment of hernia patients. Trials in hernia repair will then be able to use this definition, and subsequent trial comparison via meta-analysis will allow researchers to investigate LOD as a predictor.

Whilst reviewing the literature and holding discussions with hernia surgeons, we suspected that LOD was not being utilised in any standardised fashion. Supporting this observation, articles have suggested that written definitions of LOD are inconsistent [10]. Cross-sectional imaging with volumetric analysis is used increasingly to quantify LOD and researchers have noted that volumetric definitions of LOD can differ [11, 12]. If LOD is to be a useful concept, then it is clear that its definition should be standardised and applied consistently. In order to progress this, we used systematic review to identify the frequency with which different definitions of LOD were presented in the literature, and examined what these were.

## Methods

### Objectives

The primary aim of this systematic review was to investigate the range of written and volumetric definitions for LOD used in the literature and to report the frequency of each definition used.

### Reporting and registration

This systematic review was performed and reported in line with the Preferred Reporting Items for Systematic Reviews and Meta-Analyses (PRISMA) statement [13]. Ethical permission is not required by our University for systematic reviews of available primary literature.

### Inclusion criteria for studies

We aimed to identify indexed studies that used the term “loss of domain” in their methods when describing the morphology of hernia. No date limitation was used for our search. There was no limit to the manuscript type, allowing for the inclusion of both the primary and secondary literature in our review. Only articles written in English were included.

### Target condition

The target condition was hernia with LOD. The term LOD is frequently caused to describe large ventral hernias; however, deliberately, our search strategy did not exclude any specific subtypes or aetiologies of hernia (e.g. large inguinal or diaphragmatic hernias) as our aim was to investigate all definitions of LOD, which can be applied to hernia irrespective of hernia aetiology. We wished to encompass definitions used not only by specialist abdominal wall surgeons but also those used by general, trauma, plastic, transplant, bariatric, and paediatric surgeons.

### Participants

Participants were defined as those with large hernia with LOD, either as part of a primary study or as part of a secondary narrative review or editorial. We included paediatric patients, as the literature commonly describes the surgical repair of gastroschisis and omphalocele using the term loss of domain.

### Search strategy and string

The primary researcher, SGP, searched the PubMed database with no date limitation. Filters were applied limiting the search to “human studies”. Our search string used the keywords; “loss of domain”, “loss of abdominal domain”, and “hernia”. These terms were combined as two criteria to identify relevant articles:

1. “Loss of domain” OR “Loss of abdominal domain”
  AND
2. “hernia”

MESH terms were not used as “loss of domain” is indexed under multiple terms. After entering the above keywords, our search strategy was transformed to search for articles indexed under any mesh term containing the keyword “hernia” combining this with the keywords “loss” and “domain” (our search string is shown in “Appendix 1”).

### Citation management and screening

Identified citations were entered into a spreadsheet (Microsoft Excel for Mac 2011 v.14.5.9, Microsoft Corporation, Washington) and uploaded subsequently into a reference manager able to access the online original articles directly (Mendeley Desktop v 1.17.13, London, UK). After the search filters were applied and duplicates excluded, the citation titles were screened by two researchers (SGP, SB). Citations were excluded that were clearly unsuitable for full-text assessment. Where there was uncertainty between the two researchers for citation inclusion, differences were discussed by face-to-face discussion. The full-text of the remaining articles was assessed for eligibility, and articles were excluded if they were not written in English, not describing abdominal loss of domain, and if they were unavailable (even after using our institution’s inter-library loan service).

### Data extraction

Two researchers, SGP and SB, reviewed the full text of each article selected independently. Any data discrepancies were discussed face-to face, and if persistent they were discussed with a senior researcher. Data were extracted into an excel spreadsheet. Data extracted related to study type, year and country of publication and surgical specialty (our classification for abdominal wall specialist surgeons is shown in “Appendix 2”). Our primary aim was to extract definitions for LOD used in the literature. Our anecdotal experience was that authors used the phrase “loss of domain” as a concept to describe large hernias but without precise definition. However, any reported written and/or volumetric definitions were extracted. Free text space was also available to record any additional features regarding an individual study’s definition of LOD. Where documented, we also collected authors’ opinions of the “cut-off” threshold or percentage proportion above which they believed LOD became clinically significant, i.e. the point at which closing the abdomen becomes very challenging and physiological complication increasingly likely.

We deemed that studies originating from the same research group were acceptable as groups may use a different definition of LOD as the literature evolves. This also applied to studies who reported overlapping patient groups since our review concentrates on definitions rather than treatment effects.

### Risk of bias

We did not assess risk of bias because we were interested in definitions of loss of domain rather than methodological quality.

## Results

**Fig. 1:**
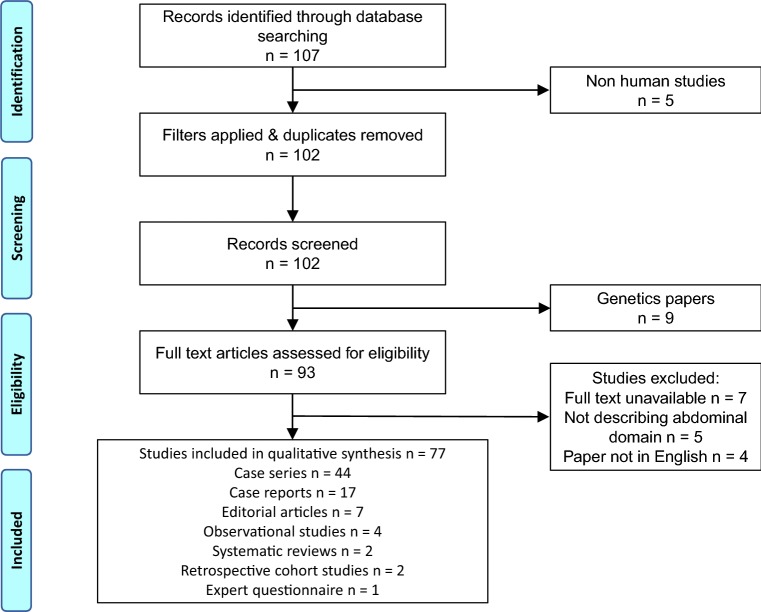
PRISMA flow chart of study selection

Our initial search retrieved 107 results (Fig. 1). After applying search filters and removing duplicates, we excluded a further 5 non-human studies, leaving 102 records for title and abstract review. After title screening, we excluded a further 9 genetics studies, leaving 93 articles for full-text review. A further 16 studies were excluded during this final stage, 7 articles couldn’t be found despite attempts to obtain them using our University’s inter-library loan service, 5 articles didn’t describe LOD, and 4 articles were not written in English; leaving 77 articles for inclusion in the systematic review (Online supplementary resource 1).

The majority of the articles, 39, originated from the USA; five were from France [11, 14–17], four from the UK [12, 18–20], and two from Italy [21, 22], India [23, 24], and Brazil [25, 26]. Six manuscripts were published prior to 2000 [27–32], 20 were published between 2000 and 2009, and 51 were published from 2010 onwards. Sixty-five articles described LOD in the context of ventral hernia patients, 9 articles described LOD caused by giant inguinal hernia, and 3 articles described giant diaphragmatic hernia. Sixty-seven articles were from the primary literature, comprising 44 case series, 17 case reports, 4 retrospective database analyses [18, 33–35], and 2 retrospective interventional studies [36] [30]. Ten articles were from the secondary literature comprising seven editorials, two systematic reviews [20, 37] and one consensus questionnaire [11]. The primary literature reported a total of 1528 patients; 419 of these were retrospective database analyses.

Thirty-eight of the articles were written by abdominal wall specialists, 16 articles were written by general surgeons, seven by paediatric surgeons, six by trauma surgeons, six by plastic surgeons, and two, one, and one by Transplant [38, 39], vascular [40] and bariatric [41] surgeons, respectively. Twenty-eight [36%] of articles presented a written definition for LOD (Online supplementary resource 2), meaning that the remaining 49 [64%] articles used the phase “loss of domain” as a concept without definition. The written definitions reported were inconsistent. Definitions varied but could be categorised into six groups (Table 1). Four out of these six groups used definitions based around four theoretical concepts. Four articles defined LOD by describing a hernia as so large that “the herniated organs have lost their right of domain inside the abdominal cavity” [15, 16, 29, 35]. Six articles use the principle of lateral contraction of the abdominal wall muscles leading to a reduced volume of the abdominal cavity and progressive visceral protrusion [39, 42–46]. Five articles use the concept of the hernia sac being a “second abdomen” and included the argument that restoring the hernia sac back into the abdominal cavity would create physiological disturbances and complications [14, 19, 25, 47, 48]. Lastly, five articles describe LOD as a large irreducible hernia containing abdominal viscera residing outside the abdominal cavity and adherent to the hernial sac [11, 26, 49–51]. Six of the definitions were miscellaneous [52–57], and two of the articles were editorials [10, 12], which highlighted inconsistencies when defining LOD. Twenty-three of the 28 [82%] definitions were reported in articles written by abdominal wall specialists. After categorising results by reporting specialty, the definitions remained inconsistent and were not dependent on the reporting surgical specialty (Table 1).

**Table 1:** The frequency of the concepts used to define “loss of domain”. Also broken down into the reporting specialties

Specialty	Loss of the “right of domain”	Contraction of the lateral abdominal wall muscles leading to reduce volume of the abdominal cavity	The concept of a second abdomen	Chronic large irreducible hernia	Miscellaneous	Editorial/literature review detailing multiple definitions	Total
AWR specialists	3 [15, 16, 35]	3 [42, 44, 46]	5 [14, 19, 25, 47, 48]	5 [11, 26, 49–51]	5 [52–54, 56, 57]	2 [10, 12]	23
General Surgeons	1 [29]	–	–		1 [55]	–	2
Plastics	–	1 [45]	–	–	–	–	1
Transplant	–	1 [39]	–	–	–	–	1
Trauma	–	1 [43]	–	–	–	–	1
Total	4 [15, 16, 29, 35]	6 [39, 42–46]	5 [14, 19, 25, 47, 48]	5 [11, 26, 49–51]	6 [52–57]	2 [10, 12]	28

Volumetric definitions used for LOD were also inconsistent. In total, 20 studies used cross-sectional imaging combined with volumetric analysis pre-operatively (Table 2). Eight studies [11, 25, 26, 44, 46, 51, 58, 59] reported the ratio of the hernia sac volume (HSV) to the abdominal cavity volume (ACV), commonly referred to as the Tanaka method [25]. Five studies [14–16, 49, 50] reported the ratio or percentage of the HSV to the total peritoneal volume (TPV = HSV + ACV), known as the Sabbagh method [15]. Four of the papers describe volumetric analyses but were unclear how LOD was calculated [19, 48, 56, 60]. Finally, 2 studies calculated HSV and ACV but simply stated these two volumes without using a ratio or a proportion [35, 61]. One editorial review discussed both methods used to calculate LOD [12]. Only two studies [59, 61] using volumetric analysis were not reported by abdominal wall specialists. Therefore, a volumetric definition for LOD remained inconsistent even amongst hernia specialists (Table 2). Fifteen papers also reported a threshold at which they believed LOD became clinically significant, but this appeared anecdotal in all, based on clinical expertise rather than any independent research. Values ranged from 10% [60] to 50% [49], with the most frequently reported value being 20% [15, 51, 58, 59].

**Table 2:** The frequency of the volumetric techniques used to define “loss of domain”; also broken down into the reporting specialties

Specialty	Tanaka et al.: ratio of the hernia sac volume/abdominal cavity volume	Sabbagh et al.: percentage of the hernia sac volume/total peritoneal volume	Unclear: Tanaka or Sabbagh	Both described	Other	Total
AWR specialists	7 [11, 25, 26, 44, 46, 51, 58]	5 [14–16, 49, 50]	4 [19, 48, 56, 60]	1 [12]	1 [35]	18
General Surgeons	1 [59]	–	–	–	–	1
Paediatric Surgeons	–	–	–	–	1 [61]	1
Total	8 [11, 25, 26, 44, 46, 51, 58, 59]	5 [14–16, 49, 50]	4 [19, 48, 56, 60]	1 [12]	2 [35, 61]	20

## Discussion

Prior to discussing the findings of this review, it is necessary to recap the complex processes that underpin large ventral hernia disease. After the linea, alba is divided by a midline hernia forms, and over time the abdominal muscles retract laterally (due to mechanical unloading), and the hernia gradually enlarges. Due to disuse atrophy, irreversible muscular fibrosis follows, the muscles becoming stiffer and less elastic [10]. Consequently, the abdominal strap muscles become shorter and thicker. These anatomical changes have physiological side effects. As intra-abdominal viscera herniate out of the abdominal cavity, intra-abdominal pressure reduces causing diaphragmatic descent and respiratory dysfunction. Portal venous stasis often occurs, causing mesenteric and bowel wall oedema, swelling the contents of the hernia sac making reduction even more challenging [26]. Venous stasis leads to congested bowel, ischaemic bowel, diarrhoea, and abdominal pain. Lastly, malalignment of the rectus muscles, atrophy of the strap muscles, and reduced intra-abdominal pressure results in an unsupported spine, precipitating chronic back pain. The pathological consequences of large ventral hernia were first described by Rives in 1973 and given the name “eventration disease” [62]. Given the clinical consequences of CVH and the difficulties of treatment, it is important that the metrics used to describe hernia morphology are relevant and consistent. Loss of domain would seem to be especially relevant, as it serves to describe the volumetric relationship between the hernia and the residual abdominopelvic cavity. However, our systematic review found that definitions are either not described or are disparate.

We found that current written definitions fell into six broad groupings. Two groups included six articles giving miscellaneous definitions [52–57] which could not categorised and two editorials listing multiple definitions [10, 12]. The remaining four groups were based on four theoretical concepts. Some articles defined LOD as the loss of the “right of domain”, a meaning that is unclear [15, 16, 29, 35]. Interestingly, “right of domain” is a phrase used in UK common law and refers to a citizen’s right to the ownership or possession of land. It is unclear how or when this phrase was used to refer to abdominal viscera; the earliest reference, we could find was from 1972. In this paper, Willard Johnson from Chelsea, Massachusetts, writes, “Infrequently a hernia is seen that has such a large sac that a significant portion of the abdominal viscera is residing outside the abdominal cavity. Over time no space is left in the abdomen to accommodate the replacement of such viscera. The contents of the sac have lost the ‘right of domain’ in the abdomen” [63]. Thus, this first definition suggests that the abdominal viscera lose the right to “belong” inside the abdominal cavity.

The second definition we identified is based on pathological processes that occur due to large abdominal defects [39, 42–46]. As described above, the abdominal strap muscles contract, shorten, and thicken. This definition uses the term “domain” to refer to abdominal cavity volume; contraction of the lateral strap muscles reduces the abdominal volume. Loss of domain, sometimes referred to as “loss of abdominal domain”, in this case really means loss of abdominal cavity volume.

Five articles used the term “loss of domain” without referring to abdominal cavity volume [14, 19, 25, 47, 48]. Perhaps the authors assume that readers are aware that “loss of domain” refers to “loss of abdominal volume”? Instead, these authors focus their definition on hernias being so large that a “second abdomen cavity” is created inside the hernia sac. Three out of these five articles [19, 25, 48] add an additional aspect to their definition mentioning the significant physiological difficulties that may occur if this “second abdomen” is reduced back into the patient’s abdominal cavity. The origins of this description of the hernia sac as a “second abdomen” are unknown.

Lastly, five articles used definitions that appeared similar or equivalent to the definition of a large irreducible ventral hernia [11, 26, 49–51]. Previous manuscripts have noticed that definitions for LOD and irreducible hernia are sometimes not dissimilar [26]. These articles use terms like, “the volume of the hernia can no longer be reduced to the abdominal cavity” [26] and, “hernia contents are set by adhesions and not reducible to the abdominal cavity” [11]. Clearly a standardised definition should distinguish hernias with LOD alone from those with irreducible and incarcerated components. Finally, it is important to mention that 49 articles, 64% of the total, use the phrase “loss of domain” without any definition at all, or any reference to a standardised definition. Consequently, we must conclude that a knowledge or understanding of the concept of LOD is often assumed by authors despite there being no standardised definition.

We believe that a globally accepted written and volumetric definition for LOD is required so that it can be used as a predictor of operative outcomes. Necessarily, this will incorporate measurements of the hernia and residual abdominopelvic cavity. However, whilst such definitions exist already, these are not consistent and are based around two equations. In the first case, the hernia sac volume (HSV) is defined as a proportion of the abdominal cavity volume (ACV) (the Tanaka method, HSV/ACV [25]). This definition was used by eight studies in our systematic review [11, 25, 26, 44, 46, 51, 58, 59]. The alternative is to describe hernia volume as a proportion of the total peritoneal volume (TPV) (the Sabbagh method, ACV + HSV = TPV, HSV/TPV [15]). This definition was used by five studies in our systematic review [14–16, 49, 50]. It is presently unclear which of these two definitions would be most appropriate or which operating surgeons feel would be the most meaningful and intelligible. The authors feel it is more logical and comprehensible to describe hernia volume as a proportion of total peritoneal volume, as this describes the percentage of abdominal viscera that has herniated.

Furthermore, a future volumetric definition of LOD may include subtypes of hernia by incorporating hernia neck width into the classification. Large hernias with narrow necks present a different surgical challenge compared to those with wide necks. In clinical practice, abdominal wall surgeons use hernia morphology to decide upon surgical approach and reconstructive techniques. Similarly, the possible array of post-operative outcomes is likely to be dependent on hernia morphology. As yet a descriptor that distinguishes between subtypes of giant hernia by neck width, or any other parameter, does not exist and future work into this is warranted.

Establishing an internationally accepted classification for LOD is the next step. To facilitate this, we intend to carry out a Delphi consensus study working with academic hernia surgeons. Several of the surgeons on the Delphi panel will be leading members of the American Hernia Society, the British Hernia Society, the European Hernia Society, and the Asian and Pacific Hernia Society. During the rounds of voting, panellists will be presented with the four written definitions and two volumetric definitions discovered by this systematic review and asked to pick their preference. Panellists will be asked to suggest improvements and alterations to their chosen definition. We will challenge panellists to establish a standardised definition that can be applied to all hernia subtypes (i.e. giant inguinal, diaphragmatic, and ventral hernias). After publication of our classification, the authors will seek endorsement from the international hernia societies to aid propagation and acceptance of this classification.

## Conclusion

Via systematic review, we have demonstrated that definitions of loss of domain are either disparate or omitted altogether. We found four broad concepts within the literature. Some were vague, and even the two volumetric definitions differed. Since loss of domain is a prime descriptor of hernia size and likely to be correlated with operative outcomes, a standardised definition is needed urgently.

### Electronic supplementary material

Below is the link to the electronic supplementary material.

Supplementary material 1 (DOCX 20 kb)

Supplementary material 2 (DOCX 95 kb)

## Appendix 1

### Search strategy and string

- (((loss of domain) OR loss of abdominal domain)) AND hernia
- Changed by the Pubmed search engine to
- ((loss[All Fields] AND (“domain”[All Fields])) AND (“hernia”[MeSH Terms] OR “hernia”[All Fields])

## Appendix 2

For the purposes of this review, we defined publications as written by abdominal wall reconstruction surgeons if the following criteria were met:

Inclusion criteria

1. The authors affiliation was to an “abdominal wall unit” or “hernia centre”.
2. Manuscripts (not case reports) published using or describing complex abdominal wall reconstructive techniques (such as pre-operative pneumoperitoneum and pre-operative Botox therapy).
3. The centre had a well-known international reputation for AWR, known to the authors of this review.
4. AWR case series published by both general surgeons and plastic surgeons.

Exclusion criteria

1. Manuscripts published by centres clearly belonging to other specialities (e.g. Paediatrics, Trauma)
2. Case reports using complex reconstructive techniques that are written by authors not affiliated to a specialist “abdominal wall unit” or a “hernia centre”
