# Supplementary material for: What Exactly is Meant by “Loss of Domain” for Ventral Hernia? Systematic Review of Definitions
Source: World J Surg. 2018 Sep 5;43(2):396–404. doi: 10.1007/s00268-018-4783-7 (PMC6329734; doi:10.1007/s00268-018-4783-7)
Supplement: Supplementary file 1 — Supplementary material 1 (DOCX 20 kb) [file 268_2018_4783_MOESM1_ESM.docx]

**Studies included in the Systematic Review**

Case reports - 17

1. Fernando EJ, Guerron AD, Rosen MJ. A case of splenic rupture within an umbilical hernia with loss of domain. Gastrointest Surg. 2015;19(4):789–91.

2. Hn D, Kumar CJ, N S. Giant inguinoscrotal hernia repaired by lichtensteins technique without loss of domain: a case report. J Clin Diagn Res. 2014;8(9):7–8.

3. Qaja E, Le C, Benedicto R. Repair of giant inguinoscrotal hernia with loss of domain. J Surg Case Rep. 2017;16(11):221.

4. Pakula A, Jones A, Syed J, Skinner R. A rare case of chronic traumatic diaphragmatic hernia requiring complex abdominal wall reconstruction. Int J Surg Case Reports. 2015;7C:157–60.

5. Obeid A, Sarhane K, Berjaoui T, Abiad F. Heterotopic intra-abdominal ossification in a complex ventral hernia defect. J Wound Care. 2014;23(2 Suppl):S5-9.

6. Suzuki T, Okamoto T, Hanyu K, Suwa K, Ashizuka S, Yanaga K. Repair of Bochdalek hernia in an adult complicated by abdominal compartment syndrome, gastropleural fistula and pleural empyema: Report of a case. Int J Surg Case Reports. 2014;5(2):82–5.

7. King J, Hayes JD, Richmond B. Repair of giant subcostal hernia using porcine acellular dermal matrix (Strattice^TM^) with bone anchors and pedicled omental flap coverage: a case report. J Med Case Rep. 2013;11(7):258.

8. Hamad A, Marimuthu K, Mothe B, Hanafy M. Repair of massive inguinal hernia with loss of abdominal domain using laparoscopic component separation technique. J Surg Case Rep. 2013;22(3).

9. Todd H, Diaz D, Roth J. Rhabdomyolysis: An unusual complication following endoscopic component separation hernia repair. J Surg Case Rep. 2012;1(9):18.

10. Berrevoet F, Martens T, Van Landuyt K, de Hemptinne B. The anterolateral thigh flap for complicated abdominal wall reconstruction after giant incisional hernia repair. Acta Chir Belg. 2010;110(3):376–82.

11. Baird R, Gholoum S, Laberge JM, Puliganda P. Management of a giant omphalocele with an external skin closure system. J Paediatr Surg. 2010;45(7):E17-20.

12. Sonmez K, Onal E, Karabulut R, Turan O, Turkyilmaz Z, Hirfanoglu I, et al. A strategy for treatment of giant omphalocele. World J Paediatr. 2010;6(3):274–7.

13. Alaish SM, Strauch ED. The use of Alloderm in the closure of a giant omphalocele. J Pediatr Surg. 2006;41(3):e37-39.

14. Serpell JW, Polglase AL, Anstee EJ. Giant inguinal hernia. Aust N Z J Surg. 1988;58(10):831–4.

15. King JN, Didlake RH, Gray RE. Giant inguinal hernia. South Med J. 1986;79(2):252–3.

16. Wartman SM, Woo K, Brewer M, Weaver FA. Management of a Large Abdominal Aortic Aneurysm in Conjunction with a Massive Inguinal Hernia. Ann Vasc Surg. 2017;42:e302-7.

17. Harrison D, Taneja R, Kahn D, Rush BJ. Repair of a massive ventral hernia in a morbidly obese patient. N J Med. 1995;92(6):387–9.

Case series - 44

18. Kingsnorth AN, Sivarajasingham N, Wong S, Butler M. Open mesh repair of incisional hernias with significant loss of domain. Ann R Coll Surg Engl. 2004;86(5):363–6.

19. Gerlach UA, Pascher A. Technical advances for abdominal wall closure after intestinal and multivisceral transplantation. Curr Opin Organ Transpl. 2012;17(3):258–67.

20. Mayagoitia JC, Suarez D, Arenas JC, Daiz de Leon V. Preoperative progressive pneumoperitoneum in patients with abdominal-wall hernias. Hernia. 2006;10(3):213–7.

21. Elstner KE, Read JW, Rodriguez-Acevedo O, Ho-Shon K, Magnussen J, Ibrahim N. Preoperative progressive pneumoperitoneum complementing chemical component relaxation in complex ventral hernia repair. Surg Endosc Other Interv Tech. 2016;1–9.

22. Dennis AJ, Salabat R, Kingsley S, Starr F, Joseph K, Wiley D, et al. Trans-abdominal wall traction as a universal solution to the management of giant ventral hernias. Plast Reconstr Surg. 2015;135(4):1113–23.

23. Bueno-Lledo J, Torregrosa A, Jimenez R, Pastor PG. Preoperative combination of progressive pneumoperitoneum and botulinum toxin type A in patients with loss of domain hernia. Surg Endosc. 2018;Feb 15.

24. Petro CC, Raigani S, Fayezizadeh M, Rowbottom JR, Klick JC, Prabhu AS, et al. Permissible Intraabdominal Hypertension following Complex Abdominal Wall Reconstruction. Plast Reconstr Surg. 2015;136(4):868–81.

25. Agnew SP, Small WJ, Wang E, Smith LJ, Hadad I, Dumanian GA. Prospective measurements of intra-abdominal volume and pulmonary function after repair of massive ventral hernias with the components separation technique. Ann Surg. 2010;251(5):981–8.

26. Martin AE, Khan A, Kim DS, Muratore CS, Luks FI. The use of intraabdominal tissue expanders as a primary strategy for closure of giant omphaloceles. J Paediatr Surg. 2009;44(1):178–82.

27. Afifi RY, Hamood M, Hassan M. The outcome of A. Double mesh intraperitoneal repair for complex ventral hernia: A retrospective cohort study. Int J Surg. 2018;53:129–36.

28. Aydinii HH, Peirce C, Aytac E, Remzi FH. A Novel Closure Technique for Complex Abdominal Wounds. Dis Colon Rectum. 2018;61(4):521–6.

29. Azar FK, Crawford TC, Poruk KE, Farrow N, Cornell P, Nadra O, et al. Ventral hernia repair in patients with abdominal loss of domain: an observational study of one institution’s experience. Hernia. 2017;21(2):245–52.

30. Daes J, Dennis RJ. Endoscopic subcutaneous component separation as an adjunct to abdominal wall reconstruction. Surg Endosc. 2017;31(2):872–6.

31. Renard Y, Lardiere-Deguette S, De Mestier L, Appere F, Colosio A, Kianmanesh R, et al. Management of large incisional hernias with loss of domain: A prospective series of patients prepared by progressive preoperative pneumoperitoneum. Surgery. 2016;160(2):426–35.

32. Punjani R, Shaikh I, Soni V. Component Separation Technique: an Effective Way of Treating Large Ventral Hernia. Indian J Surg. 2015;77(Suppl 3):1476–9.

33. Alyami M, Passot G, Voiglio E, Lundberg PW, Valette PJ, Muller A, et al. Feasibility of Catheter Placement Under Ultrasound Guidance for Progressive Preoperative Pneumoperitoneum for Large Incisional Hernia with Loss of Domain. World J Surg. 2015;39(12):2878–84.

34. Berhanu AE, Talbot SG. The “Inside-out” Technique for Hernia Repair with Mesh Underlay. Plast Reconstr Surg Glob Open. 2015;8(3):e422.

35. Cavalli M, Biondi A, Bruni PG, Campanelli G. Giant inguinal hernia: the challenging hug technique. Hernia. 2015;19(5):775–83.

36. Levy S, Tsao K, Cox CSJ, Phatak UR, Lally KP, Andrassy RJ. Component separation for complex congenital abdominal wall defects: not just for adults anymore. J Paediatr Surg. 2013;48(12):2525–9.

37. Moazzez A, Mason RJ, Darehzereshki A, Kathouda N. Totally laparoscopic abdominal wall reconstruction: lessons learned and results of a short-term follow-up. Hernia. 2013;17(5):633–8.

38. Dennis A, Vizinas TA, Joseph K, Kingsley S, Bokhari F, Starr F, et al. Not so fast to skin graft: transabdominal wall traction closes most “domain loss” abdomens in the acute setting. J Trauma Acute Care Surg. 2013;74(6):1486–92.

39. Alicuben ET, DeMeester SR. Onlay ventral hernia repairs using porcine non-cross-linked dermal biologic mesh. Hernia. 2014;18(5):705–12.

40. Zielinski MD, Goussous N, Schiller HJ, Jenkins D. Chemical components separation with botulinum toxin A: a novel technique to improve primary fascial closure rates of the open abdomen. Hernia. 2013;17(1):101–7.

41. Mangus RS, Kubal CA, Tector AJ, Fridell JA, Klingler K, Vianna RM. Closure of the abdominal wall with acellular dermal allograft in intestinal transplantation. Am J Transpl. 2012;12(Suppl 4):s55-9.

42. Sabbagh C, Dumont F, Fuks D, Yzet T, Verhaeghe P, Regimbeau JM. Progressive preoperative pneumoperitoneum preparation (the Goni Moreno protocol) prior to large incisional hernia surgery: volumetric, respiratory and clinical impacts. A prospective study. Hernia. 2012;16(1):33–40.

43. Sabbagh C, Dumont F, Robert B, Badaoui R, Verhaeghe P, Regimbeau JM. Peritoneal volume is predictive of tenison-free closure of large incisional hernias with loss of domain: a prospective study. Hernia. 2011;15:559–65.

44. Tanaka EY, Yoo JH, Rodrigues AJJ, Utiyama EM, Birolini D, Rassian S. A computerized tomography scan method for calculating the hernia sac and abdominal cavity volume in complex large incisional hernia with loss of domain. Hernia. 2010;14(1):63–9.

45. Mcadory RS, Cobb WS, Carbonell AM. Progressive preoperative pneumoperitoneum for hernias with loss of domain. Am Surg. 2009;75(6):504–8.

46. Koss W, Ho HC, Yu M, Edwards K, Ghows M, Tan A, et al. Preventing loss of domain: a management strategy for closure of the “open abdomen” during the initial hospitalization. J Surg Educ. 2009;66(2):89–95.

47. Baghai M, Ramshaw BJ, Smith CD, Fearing N, Bachman S, Ramaswamy A. Technique of laparoscopic ventral hernia repair can be modified to successfully repair large defects in patients with loss of domain. Surg Innov. 2009;16(1):38–45.

48. Ferrari GC, Miranda A, Sansonna F, Magistro C, Di Lernia S, Maggoini D, et al. Laparoscopic management of incisional hernias > or = 15 cm in diameter. Hernia. 2008;12(6):571–6.

49. Bluebond-Langner R, Keifa ES, Mithani S, Bochicchio G V, Scalea T, Rodriguez ED. Recurrent abdominal laxity following interpositional human acellular dermal matrix. Ann Plast Surg. 2008;60(1):76–80.

50. Lipman J, Medalie D, Rosen MJ. Staged repair of massive incisional hernias with loss of abdominal domain: a novel approach. Am J Surg. 2008;195(1):84–8.

51. Rodriguez ED, Bluebond-Langner R, Silverman RP, Bocchicchio G, Yao A, Manson PN, et al. Abdominal wall reconstruction following severe loss of domain: the R Adams Cowley Shock Trauma Center algorithm. Plast Reconstr Surg. 2007;120(3):669–80.

52. Ohana G, Bramnik Z, Miller A, Seror D, Ariche A, Bachar GN, et al. Treatment of large incisional abdominal wall hernias, using a modified preperitoneal prosthetic mesh repair. Hernia. 2006;10(3):232–5.

53. Kapfer SA, Keshen TH. The use of human acellular dermis in the operative management of giant omphalocele. J Paediatr Surg. 2006;41(1):216–20.

54. Lederman AB, Ramshaw BJ. A short-term delayed approach to laparoscopic ventral hernia when injury is suspected. Surg Innov. 2005;12(1):31–5.

55. Tobias AM, Low DW. The use of a subfascial vicryl mesh buttress to aid in the closure of massive ventral hernias following damage-control laparotomy. Plast Reconstr Surg. 2003;112(3):766–76.

56. Varghese TK, Denham DW, Dawes LG, Murayama KM, Prystowsky JB, Joehl RJ. Laparoscopic ventral hernia repair: an initial institutional experience. J Surg Res. 2002;105(2):115–5.

57. Carlson GW, Elwood E, Losken A, Galloway JR. The role of tissue expansion in abdominal wall reconstruction. Ann Plast Surg. 2000;44(2):147–53.

58. Coopwood RW, Smith RJ. Treatment of large ventral and scrotal hernias using preoperative pneumoperitoneum. J Natl Med Assoc. 1989;81(4):402–4.

59. Valezi AC, de Melo BGF, Marson AC, Liberatti M, Lopes AGJ. Preoperative progressive pneumoperitoneum in obese patients with loss of domain hernias. Surg Obes Relat Dis. 2018;14(2):138–42.

60. Borbely Y, Zerkowski J, Altemeier J, Eschenburg A, Kroll D, Nett P. Complex hernias with loss of domain in morbidly obese patients: role of laparoscopic sleeve gastrectomy in a multi-step approach. Surg Obes Relat Dis. 2017;13(5):768–73.

61. Oprea V, Matei O, Gheorghescu D, Leuca D, Buia F, Rosianu M, et al. Progressive preoperative pneumoperitoneum (PPP) as an adjunct for surgery of hernias with loss of domain. Chirugia (Bucur). 2014;109(5):664–9.

Observational study - 4

62. Diamond S, Cryer HG. Revising Recommendations and Outcome Measurements after Complex Open Abdominal Wall Reconstruction. Am Surg. 2015;81(10):955–60.

63. Wink JD, Wes AM, Fischer JP, Nelson JA, Stranksy C, Kovach SJ 3rd. Risk factors associated with early failure in complex abdominal wall reconstruction: a 5 year single surgeon experience. J Plast Surg Hand Surg. 2015;49(2):77–82.

64. Skipworth JR, Vyas S, Uppal L, Floyd D, Shankar A. Improved outcomes in the management of high-risk incisional hernias utilizing biological mesh and soft-tissue reconstruction: a single center experience. World J Surg. 2014;38(5):1026–34.

65. Hadad I, Small W, Dumanian GA. Repair of massive ventral hernias with the separation of parts technique: reversal of the “lost domain”. Am Surg. 2009;75(4):301–6.

Retrospective cohort study - 2

66. Sandvall BK, Suver DW, Said HK, Mathes DW, Neligan PC, Dellinger EP, et al. Comparison of Synthetic and Biologic Mesh in Ventral Hernia Repair Using Components Separation Technique. Ann Plast Surg. 2016;76(6):674–9.

67. Schnitzer JJ, Kikiros CS, Short BL, O’Brien A, Anderson KD, Newman KD. Experience with abdominal wall closure for patients with congenital diaphragmatic hernia repaired on ECMO. J Paediatr Surg. 1995;30(1):19–22.

Editorial/Review - 7

68. Halligan S, Parker SG, Plumb AA, Windsor ACJ. Imaging complex ventral hernias, their surgical repair, and their complications. Eur Radiol. 2018;

69. Patel NG, Ratanshi I, Buchel EW. The Best of Abdominal Wall Reconstruction. Plast Reconstr Surg. 2018;141(1):113e–136e.

70. Bikchandani J, Fitzgibbons RJJ. Repair of giant ventral hernias. Adv Surg. 2013;47:1–27.

71. Van Geffen HJ, Simmermacher RK. Incisional hernia repair: abdominoplasty, tissue expansion, and methods of augmentation. World J Surg. 2005;29(8):1080–5.

72. Raynor RW, Del Guercio LR. The place for pneumoperitoneum in the repair of massive hernia. World J Surg. 1989;13(5):581–5.

73. Ross SW, Oommen B, Heniford BT, Augenstein VA. Components separation in complex ventral hernia repair: surgical technique and post-operative outcomes. Surg Technol Int. 2014;24:167–77.

74. Kirkpatrick AW, Nickerson D, Roberts DJ, Rosen MJ, McBeth PB, Petro CC, et al. Intra-Abdominal Hypertension and Abdominal Compartment Syndrome after Abdominal Wall Reconstruction: Quaternary Syndromes? Scand J Surg. 2016.

Expert questionnaire - 1

75. Passot G, Villeneuve L, Sabbagh C, Renard Y, Regimbeau JM, Verhaeghe P, et al. Definition of giant ventral hernias: Development of standardization through a practice survey. Int J Surg. 2016;28:136–40.

Systematic Review - 2

76. Parker SG, Wood CPJ, Butterworth JW, Boulton RW, Plumb AAO, Mallett S, et al. A systematic methodological review of reported perioperative variables, postoperative outcomes and hernia recurrence from randomised controlled trials of elective ventral hernia repair: clear definitions and standardised datasets are needed. Hernia. 2018;22(2):215–26.

77. Janis JE, O’Neill AC, Ahmad J, Zhong T, Hofer SO. Acellular dermal matrices in abdominal wall reconstruction: a systematic review of the current evidence. Plast Reconstr Surg. 2012;130(5 Suppl 2):183s–93s.
