# Supplementary material for: What Exactly is Meant by “Loss of Domain” for Ventral Hernia? Systematic Review of Definitions
Source: World J Surg. 2018 Sep 5;43(2):396–404. doi: 10.1007/s00268-018-4783-7 (PMC6329734; doi:10.1007/s00268-018-4783-7)
Supplement: Supplementary file 2 — Supplementary material 2 (DOCX 95 kb) [file 268_2018_4783_MOESM2_ESM.docx]

| **Title** | **Study type** | **Written definition** |
| --- | --- | --- |
|  |  |  |
| **Concept of "right of domain" – 4 papers** | |  |
|  |  |  |
| Coopwood et al[29] | Case series | Loss of the "right of domain" |
| Sabbagh et al[15] | Case series | The hernia contents are held in place by adhesions and cannot be re-itegrated into the abdominal cavity [i.e. the herniated organs have lost their "right of domain" in the abdomen. |
| Sabbagh et al[16] | Case series | The hernias contents are held in place by adhesions and cannot be reintegrated into the abdominal cavity [i.e. the herniated organs have lost their "right of domain" in the abdomen]. |
| Hadad et al [35] | Retrospective analysis of database | The abdominal 'right of domain'. This often used, but poorly defined term describes the patient with a massive hernia, in which primary repair has a high chance of leading to pulmonary and/or circulatory compromise. |
|  |  |  |
| **Contraction of the lateral abdominal wall muscles leading to reduced volume of the abdominal cavity – 6 papers** | | |
|  |  |  |
| Agnew et al[42] | Case series | The term purports that the space inside the abdomen formerly occupied by the herniated viscera is forfeited over time. Irreversible decreases in abdominal muscle elasticity occurring as a result of mechanical disuse atrophy, as well as diaphragm descent are the cause of the loss of domain. The diagnosis of loss of abdominal domain does not require the hernia to be greater than a specific volume. iIt is a clinical diagnosis made by the surgeon who deems that abdominal wall compliance is insufficient for the hernia contents to be reduced and the defect to be repaired without an intolerable increase in intra-abdominal pressure. |
| Mangus et al[39] | Case series | Retraction of the abdominal wall with a loss of total volume in the peritoneal cavity |
| Dennis et al[43] | Case series | Domain loss describes this concept when applied to the lateral contractures of the abdominal wall |
| Bikhchandani et al[44] | Editorial Review | Lack of viscera in the abdominal cavity causes a decrease in the abdominal wall muscle elasticity, abdominal wall muscular atrophy, and reduced volume because of disuse. |
| Tobias et al[45] | Case series | Loss of domain: lateral migration of the rectus abdominis muscles in conjunction with flank muscle contraction leads to a progressive decrease in the volume of the abdominal cavity and worsening protrusion of the viscera. |
| Oprea et al[46] | Case series | In some instances, it is more important the volume of the exteriorized viscera and in those instances we are talking about “hernias with loss of domain”. Loss of domain by lateral musculo-aponeurotic retraction, relaxation of the diaphragm and frequent association of obesity and chronic cardio-respiratory diseases turns the patient into a biological and social invalid. |
|  |  |  |
| **The concept of the second abdomen – 5 papers** | |  |
|  |  |  |
| Kingsnorth et al[19] | Case series | This loss of domain [residence] implies that a proportion of the abdominal contents reside permanently [in a hernia sac = the second abdominal cavity] outside their natural compartment, and returning these contents will require significant physiological adaptation [mainly respiratory] if the volume exceeds > 15_20% of this compartment. |
| Tanaka et al[25] | Case series | This loss of domain means that the herniated viscera of the abdominal content inhabit, in a permanent way, the hernia sac, which behaves like a second abdominal cavity. Restoring the hernia sac contents to the abdominal cavity may lead to respiratory and circulatory disturbances. |
| Renard et al[14] | Case series | In patients with a giant incisional hernia with loss of domain, the herniated organs cannot be restored to the abdominal cavity and thereby create a "second abdomen". |
| Van Geffen et al[47] | Editorial Review | Loss of domain: in which some of the intraabdominal organs in a hernial sac form a "second abdominal cavity" and complete reduction of the hernial contents is impossible regardless of the size of the defect. |
| Berrevoet et al[48] | Case report | Loss of domain implies that a proportion of the abdominal content resides permanently outside its natural compartment, in the hernia sac, which acts as a second abdominal cavity. Returning these contents will require significant physiological adaptation [mainly res- piratory] if the volume exceeds > 15-20% of this compartment. |
|  |  |  |
| **Chronic large irreducible hernia – 5 papers** | |  |
|  |  |  |
| Valezi et al[26] | Case series | The volume of the hernia can no longer be reduced to the abdominal cavity, constituting the so called loss of domain hernias. |
| Mcadory et al[49] | Case series | Patients with 'loss of domain' have chronically herniated abdominal contents residing outside the abdominal cavity.  The diagnosis of hernia with loss of domain was established if on physical examination there was a significant amount of herniated contents outside the abdominal cavity that could not be reduced with the patient in the supine position. |
| Passot et al[11] | Expert questionnaire | Hernia contents set by adhesions and not reducible to the abdominal cavity |
| Azar et al[50] | Case series | Loss of domain occurs when an abdominal wall defect progresses to a size at which it may no longer accommodate the viscera, leading to protrusion outside the abdominal wall and into the hernia sac |
| Bueno-Lledo et al[51] | Case series | Loss of domain occurs when an abdominal wall defect progresses to a size at which it may no longer accommodate the viscera, leading to protrusion outside the abdominal wall and into the hernia sac |
|  |  |  |
| **Miscellaneous – 6 papers** |  |  |
|  |  |  |
| Zielinski et al[52] | Case series | Inability to primarily close the fascia after laparotomy |
| Baghai et al[53] | Case series | Loss of domain defines the inability of the abdominal cavity of fully accommodate the abdominal contents within its fascial boundaries. Laparoscopic LOD is the inability of the abdomen to keep the visceral contents within it whilst being insufflated with CO2. |
| King et al[54] | Case report | Majority of the patients abdominal contents were outside the abdominal cavity |
| Hamad et al[55] | Case report | The difficulty of returning herniated viscera to an abdominal cavity accustomed to being empty |
| Fernando et al[56] | Case report | "loss of domain" typically refers to a hernia in which greater than 15-20% of the intra-abdominal contents reside outside of the abdominal cavity proper. |
| Mayagoitia et al[57] | Case series | All patients had hernias with loss of domain, in which the volume of the sac and visceral contents was greater than the capacity of the abdominal cavity.  These hernias are said to have ‘‘loss of domain’’ because the contents of the hernia exceed the capacity of the abdominal cavity. |
|  |  |  |
| **Editorial/Reviews – 2 papers** | |  |
|  |  |  |
| Kirkpatrick et al[10] | Editorial Review | Multiple referenced definitions for loss of domain |
| Halligan et al[12] | Editorial Review | The ratio of the hernia sac volume to the residual abdominopelvic cavity OR describes the extent to which the abdominal cavity has lost volume to the hernia |
